# Chaperoning the *Mononegavirales*: Current Knowledge and Future Directions

**DOI:** 10.3390/v10120699

**Published:** 2018-12-08

**Authors:** Victor Latorre, Florian Mattenberger, Ron Geller

**Affiliations:** Institute for Integrative Systems Biology (I2SysBio), Universitat de Valencia-CSIC, 46980 Valencia, Spain; Victor.Latorre-Rosello@uv.es (V.L.); Florian.Mattenberger@uv.es (F.M.)

**Keywords:** *Mononegavirales*, chaperones, antivirals, Hsp70, Hsp90, CCT, respiratory syncytial virus, measles virus, mumps virus, rabies virus, Ebola virus

## Abstract

The order *Mononegavirales* harbors numerous viruses of significant relevance to human health, including both established and emerging infections. Currently, vaccines are only available for a small subset of these viruses, and antiviral therapies remain limited. Being obligate cellular parasites, viruses must utilize the cellular machinery for their replication and spread. Therefore, targeting cellular pathways used by viruses can provide novel therapeutic approaches. One of the key challenges confronted by both hosts and viruses alike is the successful folding and maturation of proteins. In cells, this task is faced by cellular molecular chaperones, a group of conserved and abundant proteins that oversee protein folding and help maintain protein homeostasis. In this review, we summarize the current knowledge of how the *Mononegavirales* interact with cellular chaperones, highlight key gaps in our knowledge, and discuss the potential of chaperone inhibitors as antivirals.

## 1. The Mononegavirales

The viral order *Mononegavirales* is comprised of eight families, of which four regularly cause human diseases: the *Filoviridae*, which includes the highly lethal Ebola (EBOV) and Marburg (MARV) viruses; the *Paramyxoviridae*, which includes measles (MeV), mumps (MuV), and parainfluenza viruses (PIV); the *Pneumoviridae*, which includes the common respiratory pathogens respiratory syncytial virus (RSV) and metapneumovirus (MPV); and *Rhabodoviridae*, which includes rabies virus (RABV) that can cause fatal encephalitis in >95% of untreated cases [1]. Viruses from this order impose significant global morbidity and mortality, and include some of the most infectious (MeV), most lethal (EBOV and RABV), and most common (RSV) viruses. In addition to causing well-established infections in human populations, emerging pathogens, such as the Nipah and Hendra viruses [2], as well as viruses causing reoccurring outbreaks, such as EBOV [3], illustrate the relevance of this virus order to human health. While effective vaccines are licensed for some *Mononegavirales* (e.g., MeV, MuV, RABV), most lack vaccines, and no effective antivirals are available. Hence, there is a pressing need for a better understanding of the biology underlying the replication of the *Mononegavirales* in order to develop novel therapeutics.

While highly diverse, the *Mononegavirales* share many common features. The genome of all members is comprised of a non-segmented, linear, negative-strand RNA of up to ~19 kb [4,5]. It is non-infectious, requiring the co-delivery of viral proteins to initiate infection, and lacks a cap structure, linked proteins, or polyadenylation. A 3′ leader (le) sequence and a 5′ trailer (tr) sequence flank the genome and regulate transcription and replication. The genomes encode 5–10 genes, arranged in individual transcription units, and transcribed viral mRNAs are both capped and polyadenylated [4]. As transcription initiates exclusively at the 3′ end of the genome and is sequential, a 3′-to-5′ expression gradient is observed. Gene order is well conserved among the *Mononegavirales*, reflecting a need for stoichiometric expression of particular proteins. While most genes give rise to a single product, up to three proteins can be produced from some genes depending on the viral family [4].

All *Mononegavirales* encode a set core of structural genes: a nucleocapsid protein (N or NP in the *Filoviridae*), an RNA-dependent RNA polymerase (L), an essential polymerase cofactor (P or VP35 in the *Filoviridae*), a matrix protein (M or VP40 in the *Filoviridae*), and envelope glycoproteins [5]. The N/NP proteins are RNA binding proteins that encapsidate viral RNA, each subunit binding several RNA bases (6–15 depending on the virus), resulting in thousands of N subunits encapsidating each genome and protecting it from degradation [6]. The L proteins are unique among both viruses and hosts, being large (>2000 amino acids), multi-domain proteins that perform RNA-dependent RNA polymerization, as well as 5′ capping, and 3′ polyadenylation of viral mRNAs [7]. The P/VP35 proteins act as essential polymerase cofactors that bridge the interactions between the L and N proteins, and also chaperone monomeric N/NP proteins, preventing their self-association and non-specific binding to cellular RNA [6,8]. Together with viral RNA, these three proteins (N, P, and L) form the nucleocapsid (NC), a ribonucleoprotein complex that mediates RNA transcription and replication, and forms the core of the virion. The M/VP40 proteins line the inside of the viral envelope and coordinate binding of viral glycoproteins with the NC in the virion [9]. Finally, the membrane glycoproteins encoded by *Mononegavirales* mediate binding to the cellular receptors and fusion of the viral envelope with cellular membranes to deliver the NC into the cytoplasm [5]. Depending on the viral family, these functions can be encoded by a single glycoprotein (G; filoviruses and rhabdoviruses), or can be split between two different proteins, a receptor binding glycoprotein (termed glycosylated (G), hemagglutinin (H), or hemagglutinin/neuraminidase (HN), depending on the virus family) and a fusion (F) glycoprotein [5].

Aside from these common core proteins, several additional structural and non-structural proteins are encoded by some *Mononegavirales*. The different structural proteins include essential transcription enhancers (M2-1 in pneumoviruses and VP30 in filoviruses), a minor matrix protein in filoviruses (VP24), and an integral membrane protein in pneumoviruses and some paramyxoviruses (SH) [5]. As all viruses must combat cellular antiviral defenses, some *Mononegavirales* further encode dedicated non-structural proteins that interfere with antiviral mechanisms, such as the NS1 and NS2 proteins of RSV, and the C and V proteins of certain paramyxoviruses. However, it is important to note that viral proteins are multi-functional, and even structural proteins can play a role in modulating host responses [10]. Hence, with these 5–11 proteins, *Mononegavirales* succeed in usurping the host cell to replicate their genomes, disarm host immune mechanisms, and successfully transmit from host to host.

Devising novel antiviral approaches requires a detailed understanding of viruses and their interaction with the host. Due to the paucity of proteins encoded by the *Mononegavirales* (and RNA viruses in general), it is not surprising to find that these viruses utilize a great number of host cell processes for successful replication. Moreover, studies have shown a selective interaction with cellular proteins that comprise central hubs within pathways, allowing for few viral proteins to coordinate large changes in cells [11,12].

## 2. Protein Folding, a Common Challenge for Both Viruses and Their Hosts

One of the most complex tasks faced by cells and viruses alike is the successful production of correctly folded, functional proteins and protein complexes. This is because reaching the correctly folded structure is essential for functionality, yet a vast number of possible conformations can be adopted by any given polypeptide chain. The challenge of protein folding is exacerbated by the fact that newly synthesized proteins emerge from ribosomes in a linear fashion, exposing hydrophobic, aggregation-prone regions that are normally buried inside the protein core during a relatively prolonged period of time (the translation rate in mammalian cells is ~20 s for a 100 amino acid protein [13]), promoting misfolding and aggregation. Furthermore, folding may be delayed until complete domains are synthesized or, for oligomeric proteins, until assembly into protein complexes. All of these events take place in a highly crowded environment [14] that favors misfolding and aggregation. Finally, cellular stress conditions, such as elevated temperatures or oxidation, can further promote incorrect folding [15].

Not only is protein folding complex, but errors in this process can have grave consequences for cells, resulting in a loss of function of the misfolded protein and the formation of toxic aggregates [15]. In addition, as ~10% of the energy in cells is consumed by translation [16], errors in protein folding can be energetically costly. To avoid potentially dangerous and costly errors in protein folding, cells are equipped with an extensive network of cellular proteins, the molecular chaperones, which oversee protein folding and maintain proteostasis. The network of chaperones and co-chaperones that comprise the chaperome is estimated to include >340 proteins in human cells [17], and constitutes ~10% of total cellular protein mass under normal conditions [18].

Several features of RNA viruses may render them particularly likely to depend on chaperones for successful replication. First, limited by their genetic coding capacity, RNA viruses encode few proteins that coordinate an astounding array of functions; this likely necessitates unique and complex structural features. Indeed, viral proteins have been shown to have distinct biochemical properties [19]. Second, RNA viruses hijack the cellular machinery to rapidly produce high levels of very few viral proteins, potentially saturating the particular folding pathways that are required for their maturation. Third, viruses rely heavily on the formation of multimeric complexes that are comprised of thousands of proteins, such as the NC or the virion matrix network. Finally, the replication of RNA viruses is extremely error-prone and thus, it generates an array of mutant proteins [20]; as most mutations are destabilizing for folding [21], a hyper-dependence on chaperones for maintaining the functionality of such proteins is likely. Hence, it is not surprising to find that viruses have evolved to universally employ chaperones for the folding of their proteins. However, despite the numerous studies that have been carried out to date on virus–chaperone interactions, the role of a relatively small fraction of the chaperome has been investigated. In this review, we provide a brief summary of the human chaperome and the current state of the knowledge of how members of the *Mononegavirales* interact with these key cellular factors, highlighting gaps in our knowledge. Finally, we discuss the potential of chaperone inhibitors as antiviral drugs.

## 3. The Human Chaperome

**Figure 1:**
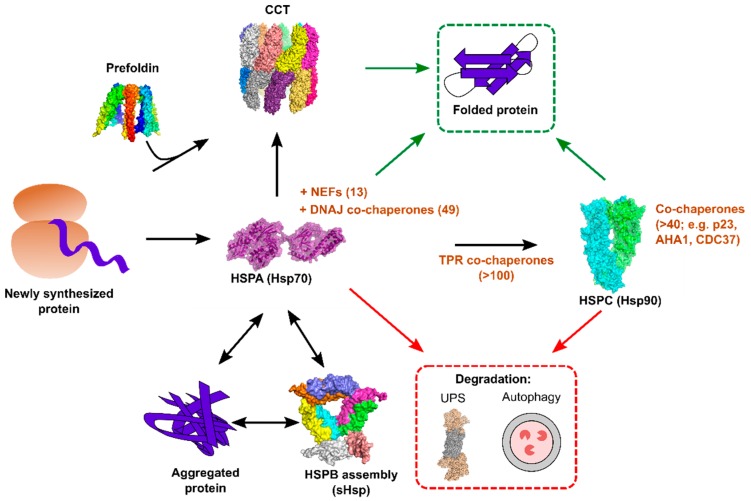
Overview of the major cytoplasmic chaperone systems in human cells. Newly translated proteins interact cotranslationally with HSPAs/Hsp70s, which function together with 49 co-chaperones of the DNAJ family and 13 nucleotide exchange factors (NEFs). Client proteins can either complete their folding via interaction with Hsp70s (green arrows) or be transferred to downstream chaperone systems. Certain client proteins require further interaction with HSPC/Hsp90, and numerous tetratricopeptide repeat (TPR) domain co-chaperones help bridge these two chaperone systems. As for Hsp70, Hsp90 function is critically dependent on a large number of co-chaperones. The chaperonin containing tailless complex polypeptide 1 (CCT) can either act downstream of Hsp70 to fold proteins, or it can receive client proteins from its co-chaperone, prefoldin. Under conditions of aberrant folding or cellular stress, protein aggregates can form in the cell, and these are refolded by Hsp70, together with small heat shock proteins (HSPB/sHsps). Finally, both Hsp70 and Hsp90 can direct proteins towards cellular degradation pathways for their disposal (red arrows). Structures were produced using PyMol version 3.5.2, and they are: Hsp70 (PDB: 2KHO), Hsp90 (PDB: 2O1V), CCT (PDB: 3IYF), sHSP (PDB: 1SHS), Proteasome (PDB: 5GJR).

The task of the chaperome is to oversee the correct folding of newly synthesized proteins, attempt to refold misfolded or aggregated proteins, and to interface with cellular degradation pathways to maintain proteostasis, including the ubiquitin–proteasome system (UPS) and autophagy [15,22,23,24]. This is accomplished by a set of relatively few ATP-dependent core chaperones, together with a large network of co-chaperones that provide client specificity, regulate chaperone activity, and provide links to different proteostasis components. Many chaperones are upregulated under conditions of stress to help maintain proteostasis, and failure to do so can result in apoptosis, directly coupling chaperone function to cell viability [25,26,27,28]. Below is a brief summary of the human chaperome (Figure 1).

### 3.1. The HSPA Chaperone Family

The HSPAs, better known as Hsp70s and Hsc70, comprise some of the most conserved and ubiquitous chaperones. These exist in all cellular compartments where ATP is found, and mediate a staggering number of cellular processes, including assisting the folding of newly translated and stress-denatured proteins, protein translocation into organelles, protein complex formation, uncoating of clathrin vesicles, as well as supporting the degradation of misfolded or aggregated proteins [28,29,30,31]. Humans encode 13 HSPA gene products, including HSPA5 (BiP) and HSPA13 in the endoplasmic reticulum (ER), the mitochondrial HSPA9 (Grp75), and numerous cytoplasmic members that are either stress-induced (Hsp70s) or constitutively expressed (Hsc70). These share a similar domain organization, with an N-terminal ATP binding and hydrolyzing domain, and a C-terminal substrate binding domain, which are connected by a hydrophobic linker [28]. In addition, HSPAs can have N-terminal localization motifs, as well as variable C-terminal sequences that mediate co-chaperone binding. HSPAs recognize short protein stretches that are enriched in hydrophobic residues that are typically buried in protein cores [32]. The HSPA chaperone cycle is dependent upon interaction with co-chaperones, nucleotide exchange factors (NEFs), and ATP [28,29]. In the ATP-bound state, the interaction of HSPAs with clients is characterized by low affinity and fast exchange rates; in contrast, in the ADP bound state, HSPAs show high affinity and slow exchange rates. Client protein delivery is mediated by DNAJ domain containing co-chaperones (DNAJs or Hsp40s), which transfer the client to ATP-bound HSPA and stimulate ATP hydrolysis [33], yielding a stable complex of ADP-bound HSPA with the client protein. Completion of the cycle is facilitated by specific NEFs, which stimulate the exchange of ADP for ATP, resulting in client release [34]. 

The complexity of the HSPA chaperone system stems from the large number of co-chaperones that regulate its ATPase activity and client binding, and which provide a link to other cellular proteostasis systems. For example, there are 49 DNAJ co-chaperones in humans. These share a conserved J domain that binds HSPAs and that stimulates ATP hydrolysis [33], but are otherwise divergent, allowing for: the binding and delivery of a wide range of clients to the HSPAs, networking with distinct chaperone systems (e.g., HSPB/small heat shock proteins and HSPC/Hsp90; see below), ribosomes, clathrin-coated vesicles, the UPS, as well as autophagy, facilitating HSPA disaggregation activity, and participating in the process of protein import into organelles, and the export of misfolded proteins from the ER [23,24,30,33]. In addition to DNAJs, >100 co-chaperones with tetratricopeptide repeat (TPR) domains interact with both HSPAs and Hsp90, providing a link between these systems [17,35].

As with the DNAJ co-chaperones, NEFs also confer specialized functions to this chaperone system. Four divergent families of HSPA NEFs can be found in human cells: the bacterial homologues of GrpE (two in the mitochondria), the Hsp110 NEF family (three cytoplasmic and one in the ER), the armadillo repeats-containing NEFs (one cytoplasmic and one in the ER), and five BAG domain-containing NEFs in the cytoplasm and nucleus [34]. While all stimulate the exchange of ADP for ATP, specialized functions have been attributed to specific NEFs. For example, Hsp110 NEFs have been implicated in protein disaggregation [24], and BAG domain NEFs have been shown to play a role in protein degradation by both the UPS and autophagy [23,36]. 

### 3.2. The HSPB Chaperone Family

There are a total of 10 HSPBs (small heat shock proteins; sHsps) in humans [37], which are exclusively cytoplasmic and nuclear. These are characterized by their small size (12–42 kDa), and by the presence of a conserved alpha-crystallin domain [38]. sHsps form dynamic structures ranging from monomers to homo- and hetero-oligomeric complexes of up to 50 subunits. The assembly of sHsps is important for their function, and is regulated by post-translational modifications [37,38]. sHsps do not utilize ATP, and they are thought to act as “holdases”, binding and assembling around misfolded proteins in order to maintain them in a conformation that is competent for refolding, disaggregation, or degradation by other chaperone systems [37].

### 3.3. The HSPC Chaperone Family

The HSPCs (Hsp90s) comprise an abundant and conserved chaperone system, constituting >2.5% of the cellular protein mass under normal conditions [18]. In humans, there are a total of five HSPC genes: HSPC1 (Hsp90AA1) and HSPC3 (Hsp90AB1) exist in the cytosol along with HSPC2 (HspAA2), a shorter isoform of HSPC1 of unclear function, as well as HSPC4 (Grp94) in the ER, and HSPC5 (Trap-1) in the mitochondria [27]. These exist largely as homodimers, and share common structural features of an N-terminal ATP binding and hydrolyzing domain, a middle domain where client binding occurs, and a C-terminal domain that is responsible for dimerization, and where numerous co-chaperones bind [39].

As with the HSPAs, the chaperone cycle of HSPCs is regulated by ATP and a large array of co-chaperones [27,39]. In the nucleotide-free state, the chaperone adopts an open V conformation, with the C-terminal domains dimerized and the N-terminal domains open, allowing for interaction with client proteins. ATP binding induces structural rearrangements that result in the association of the N-terminal domains, adopting a closed structure that stimulates ATP hydrolysis. Subsequently, ADP release results in a return to the open conformation.

The cytoplasmic HSPCs (hereafter referred to as Hsp90) act post-translationally to facilitate the folding and stabilization of client-proteins. These include many key kinases, transcription factors, and steroid hormone receptors (for an updated list, see https://www.picard.ch/downloads/Hsp90interactors.pdf), and tend to be metastable, being rapidly degraded upon Hsp90 inhibition. Hsp90 binds client proteins downstream of the Hsp70 system, with co-chaperones harboring TPR domains providing a link between these two systems [35,39]. Interestingly, Hsp90 was recently shown to play a key role in supporting the protein folding of Hsp70 client proteins, breaking the Hsp70 folding cycle to allow for subsequent Hsp70 independent folding [40]. In addition to the TPR domain co-chaperones, numerous other co-chaperones regulate Hsp90 activity. These include proteins that stabilize the open conformation, favoring client loading, such as STIP1 and CDC37, AHA1, which stimulates ATP hydrolysis, and p23, which stabilizes the closed state [39]. Finally, the co-chaperone CHIP links Hsp90, Hsp70, and the UPS [39].

Of all the chaperones, the role of Hsp90 in viral replication is best studied [41,42]. This is largely due to the availability of highly specific inhibitors [26,43] which facilitate testing, whether this chaperone system is involved in the replication cycle (Table 1). In addition, the identification of viral proteins that interact with Hsp90 is aided by the fact that many client proteins are degraded upon Hsp90 inhibition, helping unmask the relevant viral protein, as well as the ability to isolate Hsp90 in complex with client proteins. To date, Hsp90 seems to be universally employed by viruses for their replication, with the exception of the picornavirus hepatitis A [44].

### 3.4. The Chaperonin CCT

Two different chaperonins exist in human cells. The type I chaperonin (Hsp60) is found in the mitochondria, together with its co-chaperone Hsp10. The type II chaperonin, the chaperonin containing tailless complex polypeptide 1 (CCT; also known as TCP-1 Ring Complex, TRiC), is found in the cytoplasm. CCT is a 1 MDa complex comprised of two back-to-back rings, each comprised of eight different subunits, which form a cavity in which folding can occur in isolation from the cytoplasmic environment [45]. Each subunit contains three domains, an equatorial domain that mediates inter-ring interactions, a middle domain that binds and hydrolyzes ATP together with the equatorial domain, and an apical domain that binds client proteins. The apical domain harbors helical protrusions that form an iris upon ATP hydrolysis, isolating client proteins or individual domains within the chaperonin cavity. CCT is essential for viability, and it is estimated to help the folding of ~5–10% of proteins [46], including the key structural proteins tubulin and actin. Client-protein delivery to CCT can be mediated by interaction with Hsp70s or the co-chaperone prefoldin [15]. In addition, CCT can also support the formation of protein complexes. Despite its essential nature and unique chaperoning mechanism, CCT has been shown to be involved in the life cycle of relatively few viruses to date. 

### 3.5. Folding in the Endoplasmic Reticulum (ER)

The ER is the site of folding for all membrane and secretory proteins [47]. As all of the Mononegavirales encode glycoproteins that are essential for their infectious cycle, these viruses must interact with ER chaperone systems. Moreover, as ER stress resulting from the accumulation of misfolded proteins can lead to the suppression of cellular translation and cell death [25], the interaction of viruses with the ER folding machinery is likely to be carefully regulated.

Nascent proteins enter the ER cotranslationally via the translocon, where they encounter a unique folding environment that is characterized by increased oxidative conditions and high calcium concentrations, helping to mimic the extracellular environment [47]. As in the cytosol, proteostasis is maintained by a set of chaperones; however, the ER lacks degradation capabilities, and therefore, misfolded proteins must be retro-translocated into the cytoplasm in a process termed ER-associated degradation (ERAD) [47,48]. Analogous systems to the cytosolic HSPA/Hsp70 and HSPC/Hsp90 systems are present in the ER to facilitate both protein folding and quality control. The ER Hsp70 system is composed of the Hsp70 BiP, five DNAJ co-chaperones, and two NEFs. It is an essential aspect of folding of all ER proteins, from their translocation into the ER, until their final maturation or their exit from the ER for degradation. The ER Hsp90 system is composed of Grp94 (HSPC4), which represents the major glycoprotein of the ER. Unlike cytoplasmic Hsp90, co-chaperones of Grp94 are poorly defined, and relatively few client proteins have been identified to depend on this chaperone for folding [47]. In addition to folding, Grp94 also plays a key role in quality control and ERAD [49].

The ER includes an additional chaperone system that acts in concert with the Hsp70 and Hsp90 systems to fold glycoproteins, the lectin-binding chaperone system [47]. It consists of the chaperones calreticulin and calnexin, *N*-glycan processing enzymes that prevent aggregation and premature export from the ER, and a UDP-glucose:glycoprotein glucosyltransferase that is involved in quality control. Additional factors, such as peptidyl-propyl isomerases and protein disulfide isomerases help in the folding and maturation of ER proteins. As in the cytoplasm, the ER chaperome is composed of an interconnected network that cooperates to regulate proteostasis [50].

## 4. Chaperone–*Mononegavirales* Interactions

Numerous studies have investigated the role of chaperones in the life cycle of the *Mononegavirales*. Below is an up-to-date summary of known chaperone–*Mononegavirales* interactions.

### 4.1. Hsp70s in the Life Cycle of the Mononegavirales

A general indication that the Hsp70s play a role in the replication of the *Mononegavirales* is provided by the induction of Hsp70 during infection with certain members of this virus order [51,52,53,54,55,56,57,58,59], and the co-localization of these chaperones to sites of viral replication (cytoplasmic inclusion bodies) in cells infected with canine distemper virus (CDV) [60], RSV [61,62], RABV [53,63], and MuV [51]. Early studies showed that pretreatment of cells with acute heat stress, which upregulates numerous chaperones, including Hsp70, increased the polymerase transcription of NC purified from cells infected with either MeV or the related CDV [64,65,66,67]. In addition, heat shock also stimulated virus production and resulted in the appearance of a large plaque phenotype [67,68], supporting a key role for chaperones. For CDV and MeV, this stimulation of transcription was shown to be directly mediated by the interaction of Hsp70 with the NC [65,69,70]. Specifically, the addition of blocking antibodies to Hsp70 reduced transcription from purified NC, while the addition of exogenous Hsp70 stimulated it [65]. Hsc70 was also shown to co-purify with isolated NC from infected cells. However, blocking antibodies to this chaperone or its addition to isolated NC had no effect on transcription, suggesting that Hsc70 does not play a direct role in NC transcription [65]. Interestingly, NC that co-purified with Hsp70 from infected cells had higher transcriptional activity than those which did not co-purify with the chaperone, even if exogenous Hsp70 was added following purification, potentially implicating additional cellular players [65]. Finally, Hsp70 overexpression leads to increased virus production in cells infected with CDV, MeV, and the rhabdovirus vesicular stomatitis virus (VSV) [67,68,69,71,72]. For the latter, Hsp70 must be expressed prior to infection in order to increase viral replication, as a recombinant VSV encoding Hsp70 did not result in increased virus production [72].

The stimulatory effect of Hsp70 on MeV NC was shown to stem from its interaction with the N protein [70]. The interaction was localized to the unstructured C-terminal domain of N [70,71,73], where the polymerase cofactor P binds N [73]. Biochemical analysis using purified proteins showed that Hsp70 alone has low affinity for the C-terminal domain of N, but the addition of the DNAJ co-chaperone DNAJB1 is sufficient to increase its ATPase activity and affinity for N [74]. DNAJB1 itself did not interact directly with N, but a ternary complex could be isolated in the presence of Hsp70 [74]. It is important to note that the relevant DNAJ protein in the context of infection has not been identified. Interestingly, a naturally occurring mutation in the C-terminal domain of the MeV N protein significantly reduces its interaction with Hsp70, as well as the ability of this chaperone to stimulate NC transcription [70,73,74,75], and strains carrying this mutation have lower fitness [75]. Hence, for MeV and CDV, Hsp70 seems to play a role in transcription by binding the N protein, potentially by regulating its interaction with P, altering its conformation in the NC, or helping to localize it to lipid rafts where transcription occurs [61].

An interaction between Hsp70 and proteins forming part of the replication complex was also described for other *Mononegavirales*. For EBOV, immunoprecipitation of NP from transfected cells was shown to co-purify Hsp70, the Hsp70 NEF BAG2, and the Hsp70 co-chaperone DNAJA2 [76]. Of these, only the interaction between DNAJA2 and NP was lost upon RNase treatment of purified NP, suggesting either a conformation-dependent interaction or direct binding to cellular RNA [76]. The relevance of these findings, however, remain to be demonstrated in the context of infection. Nevertheless, inhibition of Hsp70 using a pharmacological inhibitor was shown to reduce EBOV replication in the context of a mini-genome system, where cells are transfected with a reporter genome together with the proteins required to form the NC (NP, VP35, VP30, and L), providing evidence for Hsp70s in playing a role in the replication of this virus [76,77]. In addition to interacting with NP, Hsp70 was shown to co-purify with EBOV L and P complexes purified from insect cells [78].

A role for Hsp70 in RSV transcription has also been directly demonstrated. Inhibition of Hsp70 with either pharmacological inhibitors [79] or blocking antibodies [61] was shown to reduce RSV transcription in cell lysates. Interestingly, despite the different strategies employed, both studies demonstrated that low levels of Hsp70 inhibition actually stimulated transcription, while higher levels were inhibitory, suggesting a complex interaction. For individual NC components, the RSV L protein was shown to bind two different Hsp70 isoforms (HSPA1 and HSPA4) in a proteomic study [79], and to co-purify with RSV L and P complexes isolated from insect cells [79]. In addition, Hsp70 was found to interact with both N and P in RSV infected cells [56]. Interestingly, unlike what is observed with NC from infected cell lysates, transcription mediated by RSV P:L complexes isolated from insect cells, which co-purify with Hsp70, is not sensitive to Hsp70 inhibition, suggesting a requirement for the complete NC or additional cellular factors [79].

For MuV, the co-expression of N and P in cells is sufficient to localize Hsp70 to sites of RNA replication (inclusion bodies) [51], and a direct interaction between the MuV L protein and Hsp70 has been reported when L is expressed by itself in cells [80]. However, in contrast to MeV and RSV, MuV replication is not directly influenced by Hsp70, as either the knockdown of Hsp70 [51] or the pharmacological inhibition of this chaperone [80] did not reduce viral replication significantly. Rather, it was shown that Hsp70 knockdown increased the apoptosis of infected cells, and resulted in the accumulation of ubiquitinated P protein [51]. Hence, for MuV, Hsp70 seems to regulate P levels, which could potentially aid in preventing apoptosis. Of note, pharmacological inhibition of Hsp70 was shown to potentiate the antiviral effects of Hsp90 inhibition during MuV replication (see Section 5. Chaperone inhibitors as antivirals), and enhanced the degradation of L, indicating that Hsp70 can play a role in MuV replication under stress conditions [80].

Not only have members of the Hsp70 family been demonstrated to bind viral proteins, but an interaction with viral RNA has also been described. For RABV, Hsc70 was shown to bind the 3’ leader RNA (le) [52]. le RNA is the first RNA to be produced during infection, and is suggested to regulate replication, although its function is not well elucidated. le RNA overexpression was shown to be antiviral and reduced the binding of N to viral genomic RNA [52]. Interestingly, Hsc70 knockdown resulted in increased le RNA expression in infected cells, and this was accompanied by a reduction in viral RNA levels and virus production [52]. In addition, for EBOV, Hsc70 was shown to bind an AUUUA motif in the 5’ trailer RNA, and knockdown of Hsc70 was shown to reduce virus replication [81].

#### Hsp70 Co-Chaperones in the Life Cycle of the Mononegavirales

Less is known about the role of Hsp70 co-chaperones in the replication of the *Mononegavirales*. Aside from the abovementioned role of an unknown DNAJ protein implicated in Hsp70 interactions with the N protein of MeV [74] and NP of EBOV [76], several studies have identified DNAJ proteins in proteomic studies, yet these lack validation [11,79,82,83]. Similarly, little information is available regarding the role of different Hsp70 NEFs. The only exception is for BAG3, which has been demonstrated to play a role in the replication of the filoviruses EBOV and MARV [84]. BAG3 was identified in a screen of cellular proteins that could interact with the late domains of the VP40 matrix proteins of these viruses, which play a role in viral budding. It was found that BAG3 binds EBOV and MARV VP40, and reduces the budding of virus-like particles (VLPs) from VP40-expressing cells. Accordingly, the overexpression of BAG3 resulted in reduced VLP budding, while the knockdown of BAG3 increased it. Furthermore, BAG3 was shown to alter the localization of VP40 to aggresomes containing the autophagosomal marker LC3, a function that is in agreement with the established role of BAG3 in autophagy [36]. While the relevance of BAG3 in the context of replication of EBOV was not investigated, grafting the late domain of VP40 onto the matrix protein of VSV was used to show that BAG3 overexpression can reduce virus production. Since BAG3-mediated autophagic degradation occurs via a multi-chaperone complex containing Hsp70, Hsp40, sHsps, and CHIP [36], it is of interest to examine whether this canonical degradation pathway is relevant for filovirus replication. In sum, much work lies ahead for understanding the role of the Hsp70 system, including its numerous co-chaperones, in the replication of the *Mononegavirales*.

### 4.2. Hsp90s in the Life Cycle of the Mononegavirales

A key role for Hsp90 has been demonstrated in the replication of numerous *Mononegavirales*, largely aided by the availability of specific Hsp90 inhibitors (see Section 5 and Table 1). In general, Hsp90 has been shown to be required for chaperoning the large, multi-domain, metastable L protein [7,85]. To mediate transcription and replication, L must form a complex with P and N, and for some viruses, additional transcription enhancers (M2-1 or VP30). Its complex structure and the need for further assembly with additional factors likely render L critically dependent on Hsp90 for folding and generation of functional replication complexes.

**Figure 2:**
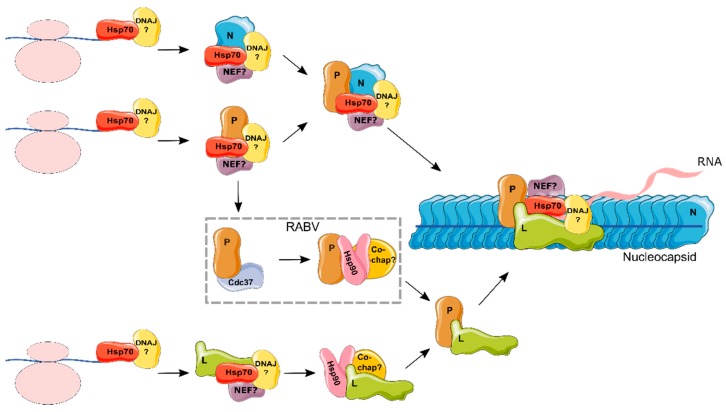
Hypothetical model for the role of chaperones in the formation of the replication and transcription complex of the *Mononegavirales*. Hsp70 has been shown to be part of the nucleocapsid (NC; demonstrated for RSV, RABV, and MeV) and to individually bind different proteins that comprise the NC: N (RSV, MeV, EBOV, and RABV), P (MuV and RSV) and L (MuV) (see text for references). As the function of Hsp70 is dependent on DNAJ proteins and NEFs, these are assumed to form part of the complex, but they remain to be defined (indicated by a question mark). Hence, it is likely that Hsp70, together with its co-chaperones, is required for the folding, assembly, or localization of N, P, and L. An intermediate complex between N, P, Hsp70, and co-chaperones prior to NC incorporation is possible. On the other hand, Hsp90 was shown to bind the viral polymerase (L; demonstrated for RSV, MeV, and MuV). Overall, Hsp90 co-chaperones remain largely undefined, with the exception of CDC37, which was shown to bind P from RABV. As Hsp90 works downstream of Hsp70, it is assumed that L binds Hsp70 prior to interacting with Hsp90. Finally, following the assembly of L with P, Hsp90 was shown to no longer be required. Elements from Servier Medical Art (https://smart.servier.com/) were used to make this figure.

Perhaps the best-described mechanism for the interaction of the replication complex with chaperones stems from work with the paramyxoviruses MeV [86] and MuV [80] (see Figure 2). These studies have shown that Hsp90 is essential during the early steps of L maturation. Specifically, L has been shown to directly bind Hsp90, with the interaction being mediated by the N-terminal domain of L in the case of MeV [86]. Interestingly, this is where P also binds L [86]. When Hsp90 is inhibited during the synthesis of L, the polymerase misfolds and is degraded [80,86]. However, if Hsp90 is present, L can fold and assemble with P, at which point the complex is rendered Hsp90-independent, as evidenced by the fact that L is no longer degraded upon Hsp90 inhibition. Furthermore, transcription by mature polymerase complexes was shown to be insensitive to Hsp90 inhibition, unlike Hsp90 inhibition during de novo polymerase synthesis [86]. Hence, Hsp90 is an essential chaperone that is required for the folding and maturation of the polymerase, but it is dispensable following the assembly of L and P, or within assembled NC.

The fate of L in cells following Hsp90 inhibition has also been investigated in these studies. L expressed in isolation is nearly completely insoluble, and the co-expression of P is required to increase its solubility [80,86]. When Hsp90 activity is blocked by pharmacological inhibitors, L that is expressed together with P (soluble L) is degraded in a manner that is independent of the proteasome [86], likely via autophagy. In contrast, L that is expressed in the absence of P (insoluble L) is ubiquitinated and degraded by the UPS [80,86] via interaction with Hsp90, Hsc70, Hsp70, and CHIP [80]. These results indicate that both the folding of L and its fate upon Hsp90 inhibition are dependent on the expression of P. As P is more abundant than L in infected cells, the relevance of L degradation by the UPS when expressed in isolation is not clear. Moreover, these results highlight the fact that care must be taken when examining the interaction of viral proteins in isolation, or as fusion proteins that may increase their solubility and potentially alter their folding and/or degradation. It is important to note that the UPS has been shown to be involved in the degradation of L following Hsp90 inhibition in the context of infection for other *Mononegavirales*, including RSV [87] and VSV [88], suggesting the fate of Hsp90-dependent viral client proteins following Hsp90 inhibition can differ from virus to virus.

#### Hsp90 Co-Chaperones in the Life Cycle of the Mononegavirales

As for the Hsp70s, very little information is available regarding the role of Hsp90 co-chaperones in the replication of the *Mononegavirales*. The only exception is for RABV, where the kinase-specific Hsp90 co-chaperone CDC37 [39] has been demonstrated to play a role in P protein maturation [89]. Specifically, this work showed that both CDC37 and Hsp90 bind the P protein independently, as mutants of CDC37 that do not bind Hsp90 could still bind the P protein. Overexpression of both Hsp90 and CDC37 increased P levels in cells by stabilizing the protein post-translationally, while their depletion by RNA interference resulted in its degradation by autophagy. Whether Hsp90 and CDC37 play a role in the formation of P and L complexes remains to be shown. By analogy to other *Mononegavirales*, the L protein of RABV may bind Hsp90, but this has not yet been demonstrated. Moreover, while the P protein of RABV binds both Hsp90 and CDC37, it is not clear if this is a general mechanism, as the MuV P protein was shown to not bind Hsp90 [80]. It is therefore of interest to investigate whether CDC37 plays a role in the replication of additional *Mononegavirales*, and to further define the role of Hsp90 co-chaperones in the life cycle of these viruses.

### 4.3. The Chaperonin CCT in the Life Cycle of the Mononegavirales

Despite the unique chaperoning mode of CCT and its essential nature, little is known about the role of this chaperone in the replication of the *Mononegavirales*. The only exception is for RABV, where it was shown that knockdown of two CCT subunits, CCTγ [90] and CCTα [91], reduces viral replication, implicating CCT as a proviral factor. Furthermore, CCTγ and CCTα were shown to localize to sites of viral replication (Negri bodies) in cells cotransfected with RABV N and P proteins [90,91]. Surprisingly, CCTβ was shown to not colocalize with N or P in Negri bodies [91], despite the fact that CCT subunits are largely found as part of the CCT chaperone complex, and a role for individual CCT subunits has been appreciated only in a few cases. However, CCTα and CCTγ, but not CCTβ, have been identified as microtubule-associated proteins in vitro [92], suggesting a possible explanation for their recruitment to Negri bodies, whose dynamics are known to be altered by microtubules [63]. Attempts to co-immunoprecipitate the viral N or P proteins with CCTα or CCTγ were unsuccessful [90,91]. Hence, the mechanisms underlying the interaction of the CCT complex or individual CCT subunits with RABV proteins remain to be fully elucidated. For other *Mononegavirales*, proteomic studies have identified individual CCT subunits to interact with the RSV L protein [79] and both the EBOV VP24 [93] and NP proteins [76], while multiple subunits were identified to interact with the MeV V protein [11]. However, as further validation was not carried out in these studies, the relevance of these associations remains unclear. 

### 4.4. Folding the Glycoproteins of the Mononegavirales in the ER

The generation of infectious particles by the *Mononegavirales* is critically dependent on successful folding of the envelope glycoproteins within the ER. Hence, it is not surprising to find that viruses that need to rapidly produce large amounts of glycoproteins employ mechanisms to increase the ER folding capacity. For the *Mononegavirales*, the induction of chaperones during infection has been documented for Sendai Virus [94], Simian virus 5 (SV5) [95], RSV [96], MeV [97], and EBOV [98]. In addition, the physical interaction of viral proteins with chaperone components has been demonstrated by co-immunoprecipitation of BiP with the glycoproteins of the paramyxoviruses SV5 (HN protein) [95,99,100], Sendai virus (HN and F proteins) [94], and MeV (H and F proteins) [97], as well as the RSV F protein [101] and RABV G protein [102]. Direct proof for a role of BiP in the replication cycle of EBOV, MeV, MuV, and RABV was provided by studies showing reduced replication following knockdown of the chaperone [98,103,104,105]. Finally, BiP overexpression was shown to augment the virulence of MeV in tissue culture, although whether this result is due to increased viral replication or cell death remains to be determined [105].

In addition to BiP, members of the lectin-binding chaperone system have also been shown to bind the glycoproteins of different *Mononegavirales*, including EBOV [106], MeV [97], RSV [96], RABV [102], and VSV [107,108]. In contrast, interactions with the ER resident Hsp90, Grp94, the most abundant glycoprotein in the ER, have not been reported for any member of the *Mononegavirales*. However, this chaperone has been implicated in the life cycle of VSV due to its role in folding Toll-like receptors that are required for infection [109].

## 5. Chaperone Inhibitors as Antivirals

No antivirals are currently approved for treating infections with the *Mononegavirales*. Moreover, the extreme evolutionary capacity of RNA viruses that stems from their high mutation rates [20], large population sizes, and short replication times can readily select for drug-resistant variants that render antivirals ineffective. Hence, novel antiviral approaches, and especially those that are refractory to the development of drug resistance, are urgently needed.

Due to the general use of chaperones by viruses, modulation of chaperone function represents an attractive antiviral strategy (see Table 1). To date, the most druggable chaperone has been Hsp90. Work using various Hsp90 inhibitors has shown that non-toxic concentrations display antiviral activity against numerous *Mononegavirales*, including: the filovirus EBOV [110]; the paramyxoviruses PIV2 [88], MeV [86,111], MuV [80], and SV5 [88]; the pneumovirus RSV [62,79,87,112]; and the rhabdoviruses RABV [89] and VSV [88]. The successful inhibition of Hsp90 in humans for cancer treatment [26,43] highlights the feasibility of antiviral approaches that target chaperones. Moreover, Hsp90 inhibitors have thus far not been shown to elicit drug resistance [87,113], suggesting these may not suffer from one of the major limitations of current antiviral approaches targeting RNA viruses.

Several different Hsp70 inhibitors have been described that target the ATP binding domain (e.g., VER155008, as well as MKT007 and its derivatives YM1 and JG40) and the substrate binding domain (pifithrin-μ). While these have been shown to reduce replication in the context of mini-genomes or other surrogates of viral replication for EBOV [76,77], RSV [79], and MPV [55], the potency of these inhibitors appears to be less than those targeting Hsp90. Indeed, for MeV and MuV, Hsp70 inhibitors did not influence replication [80]; however, despite not showing antiviral effect on its own, the Hsp70 inhibitor VER155008 was shown to strongly potentiate the antiviral activity of Hsp90 inhibitors for both viruses [80], suggesting that combinatorial chaperone inhibition may be a promising antiviral approach. 

As in the cytoplasm, inhibiting protein folding in the ER is likely to have broad antiviral activity against the *Mononegavirales* due to their general use of this compartment for the generation of essential glycoproteins. Indeed, non-specific interference with protein folding in the ER by altering calcium levels or by blocking key glycosylation enzymes that prevent folding have been reported to block the maturation of VSV [107,114], SV5 [100], MeV [97], and EBOV [115]. In addition, compounds that inhibit multiple chaperones, including those present in the ER, such as Sorafenib, OSU-0312, or epigallocatechin gallate, can reduce the replication of EBOV, MeV, and MuV infection [98,103,104,116]. Hence, targeting the ER folding machinery is likely to represent a broad-spectrum antiviral approach, but specific antivirals are largely unavailable [116].

As new compounds targeting the chaperome are discovered, the possibility of inhibiting less central nodes in the chaperone network may arise. Such antivirals may show improved toxicity profiles, as the range of off-target effects is likely to be drastically reduced compared to the inhibition of Hsp90 or Hsp70. For this, an in-depth knowledge of the relevant chaperones and co-chaperones that are involved in viral replication will be of great importance. In sum, the broad-spectrum antiviral activity of chaperone inhibitors and their apparent low rate of drug resistance make antiviral approaches focused on chaperone inhibition of interest for the treatment of viral infections. It is important to note, however, that the role of chaperones extends beyond their function in the cell. The extracellular release of chaperones has been shown to influence inflammation, immune activation, and even the outcome of infection for some *Mononegavirales* [54,71,72,117,118,119]. Hence, the ability of chaperone inhibitors to block viral replication must be evaluated in relevant models that also take into account the extracellular roles of chaperones. 

## 6. Conclusions

Despite the progress that has been made in deciphering the interactions between chaperones and the *Mononegavirales*, significant gaps in our knowledge remain. The chaperone dependence of numerous viral proteins from the *Mononegavirales* remains to be defined (e.g., SH, VP24, NS1, NS2, M2-1, M2-2, V, and C). Proteomics studies frequently identify chaperones as interacting partners [11,76,79,82,93,112,120], but many times they lack validation of the interaction and formal testing of the relevance of the identified chaperone in the context of infection. Even for well-studied interactions, such as that of the NC with Hsp70, or the L protein with Hsp90, the role of co-chaperones or upstream and downstream chaperone systems remains to be fully elucidated. For example, while a clear interaction between NC and Hsp70 has been established for MeV, the relevant DNAJ and NEF co-chaperones remain unknown. Moreover, the generality of this finding for other *Mononegavirales* is not clear. Similarly, Hsp90 has been shown to be involved in the folding of L from numerous viruses. However, Hsp90 works downstream of Hsp70/Hsc70, and thus far, an interaction between the latter and L has only been formally shown for MuV [80]. As for Hsp70, our knowledge of Hsp90 co-chaperones involved in the folding of L remains limited. Thus far, of the numerous Hsp90 co-chaperones, the only one identified to date to play a role in the replication of any of *Mononegavirales* is CDC37 for chaperoning the RABV P protein [89]. Due to the large number of co-chaperones for both Hsp70 and Hsp90, it is possible that functional redundancies may hinder the identification of individual co-chaperones involved in viral replication. In favor of this argument, a study using RSV found that knocking down two co-chaperones identified in their proteomic study to bind L, STIP1 and DNAJA2, had no effect on viral replication [79]. However, contrary to this argument, a recent study investigating the function of DNAJ proteins in the replication of dengue virus was able to identify a role for several of these co-chaperones in distinct stages of the viral replication cycle [121]. Finally, it is important to note that the role of key chaperone systems in the cells, such as the sHsps and CCT, remains undefined for nearly all the *Mononegavirales*.

While much can be learned about viral biology from studying chaperones, the opposite may be true as well. In particular, recent works have appreciated the fact that chaperones exist in dynamic multi-chaperone complexes that are likely to confer specific functions that are required to meet cellular proteostasis demands [122]. In this regard, viral infection may provide a valuable tool for the reproducible induction of alterations in the composition of chaperone complexes that can help to decipher both the basis and functional importance of such changes.

## Figures and Tables

**Table 1:** Antiviral activity of chaperone inhibitors against the *Mononegavirales*.

Family	Virus	Chaperone Target	Compound ^1^	Reference(s)
*Filoviridae*	EBOV	Hsp90	17AAG, GA, Radicicol, SNX 9503/2113/7023/7021	[110]
		Hsp70	RNA interference	[103]
			VER155008	[76]
			JG40	[77]
		DNAJB2	RNA interference	[83]
		Multiple chaperones	EGCG	[98]
		BiP	RNA interference	[83,98,103]
		Hsc70	RNA interference	[81]
	MARV	BiP	RNA interference	[83]
*Paramyxoviridae*	MuV	Hsp90	17AAG	[80]
		Hsp70	VER155008 + 17AAG	[80]
		BiP + Hsp27	RNA interference	[103]
		Multiple chaperones	Sorafenib, Sorafenib + Sildenafil, Sildenafil + AR-12	[104,105]
	MeV	Hsp90	GA, 17DMAG	[86,111]
			RNA interference	[86]
		Hsp70	VER155008 + 17AAG	[80]
		BiP + Hsp27	RNA interference	[103]
		Multiple chaperones	Sildenafil + AR-12	[105]
	PIV2	Hsp90	GA	[88]
	SV5	Hsp90	GA/Radicicol	[88]
*Pneumoviridae*	RSV	Hsp90	GA, 17AAG, 17DMAG	[62,79,87]
			RNA interference	[62]
		Hsp70	VER155008, PIF, MKT007, YM1	[79]
		Hsc70	RNA interference	[62]
	MPV	Hsp70	VER155008	[55]
*Rhabdoviridae*	RABV	Hsp90	17AAG	[89]
			RNA interference	[89]
		Hsp90/Cdc37	Celastrol	[89]
		Cdc37	RNA interference	[89]
		Hsp70	RNA interference	[53]
		CCTγ	RNA interference	[90]
		CCTα	RNA interference	[91]
		Block Hsp induction	Quercetin	[53]
		Multiple chaperones	Sorafenib, Sorafenib + Sildenafil	[105]
	VSV	Hsp90	GA	[88]
			RNA interference	[88]

^1^ Abbreviations: EBOV: Ebola virus; MARV: Marburg virus; MuV: mumps virus; MeV: measles virus; PIV2: parainfluenza virus 2; SV5: Simian Virus 5; RSV: respiratory syncytial virus; MPV: metapneumovirus; RABV: rabies virus; VSV: vesicular stomatitis virus; GA: Geldanamycin; 17AAG: 17-allyl-17-demethoxygeldanamycin; 17DMAG: 17-desmethoxy-17-*N,N*-dimethylaminoethylaminogeldanamycin; EGCG: Epigallocatechin gallate; AR-12: OSU-0312. PIF: pifithrin-μ.
